# MOST wanted: navigating the MAPK-OIS-SASP-tumor microenvironment axis in primary pediatric low-grade glioma and preclinical models

**DOI:** 10.1007/s00381-024-06463-z

**Published:** 2024-05-25

**Authors:** Romain Sigaud, Tilman Brummer, Daniela Kocher, Till Milde, Florian Selt

**Affiliations:** 1 Hopp Children’s Cancer Center Heidelberg (KiTZ), Heidelberg, Germany; 2 Clinical Cooperation Unit Pediatric Oncology, German Cancer Research Center (DKFZ) and German Consortium for Translational Cancer Research (DKTK), Heidelberg, Germany; 3 National Center for Tumor Diseases (NCT), Heidelberg, Germany; 4 Institute, of Molecular Medicine and Cell Research (IMMZ), Faculty of Medicine, University of Freiburg, Freiburg, Germany; 5 Centre for Biological Signaling Studies BIOSS, University of Freiburg and German Consortium for Translational Cancer Research (DKTK), Freiburg, Germany; 6 German Cancer Research Center (DKFZ), Heidelberg, Germany; 7 Faculty of Biosciences, Heidelberg University, Heidelberg, Germany; 8 Department of Pediatric Hematology and Oncology, Heidelberg University Hospital, Heidelberg, Germany

**Keywords:** Pediatric low-grade glioma, MAPK pathway, Oncogene-induced senescence, Senescence-associated secretory phenotype, Microenvironment

## Abstract

Understanding the molecular and cellular mechanisms driving pediatric low-grade glioma (pLGG)—the most prevalent brain tumor in children—is essential for the identification and evaluation of novel effective treatments. This review explores the intricate relationship between the mitogen-activated protein kinase (MAPK) pathway, oncogene-induced senescence (OIS), the senescence-associated secretory phenotype (SASP), and the tumor microenvironment (TME), integrating these elements into a unified framework termed the MAPK/OIS/SASP/TME (MOST) axis. This integrated approach seeks to deepen our understanding of pLGG and improve therapeutic interventions by examining the MOST axis’ critical influence on tumor biology and response to treatment. In this review, we assess the axis’ capacity to integrate various biological processes, highlighting new targets for pLGG treatment, and the need for characterized in vitro and in vivo preclinical models recapitulating pLGG’s complexity to test targets. The review underscores the need for a comprehensive strategy in pLGG research, positioning the MOST axis as a pivotal approach in understanding pLGG. This comprehensive framework will open promising avenues for patient care and guide future research towards inventive treatment options.

## Introduction

Pediatric low-grade gliomas (pLGGs), the most frequent brain tumors in children [1], present a therapeutic conundrum of benign histology but significant clinical challenge due to their high risk of multiple progressions [2] and morbidity, making them a high-burden chronic disease. While the overall survival for these patients is excellent with a 10-year overall survival (OS) of 96% [2], 10-year progression-free survival (PFS) can be poor and is below 50% for partially resected tumors [3]. Patients are furthermore burdened with life-impeding morbidities related to the tumor location and/or treatment sequelae [4, 5].

Central to this disease is the mitogen-activated protein kinase (MAPK) pathway, a critical regulator of cell fate decisions including proliferation, differentiation, and apoptosis. Dysregulation of the MAPK pathway, resulting from various molecular alterations leading to activation, is a hallmark of pLGG [6–8].

The genetics and biology of the MAPK in pLGG is well understood and has led to the translation of preclinical concepts into the clinical reality of targeted MAPK inhibition. While the preclinical work has been focusing on the tumor compartment, the multiple aspects of the biology downstream, in particular of the oncogene-induced senescence (OIS), the senescence-associated secretory phenotype (SASP), and their interconnection with the tumor microenvironment (TME) are still incompletely understood. These components need to be investigated as interactive and not as separate entities in order to meet the clinical challenges of efficacious therapies, predictive stratification, rebound, and resistance in pLGG therapy.

In this review, we propose an integration of the MAPK pathway, OIS, SASP, and the TME into a MOST axis, a framework encapsulating the multifaceted biology of pLGG. Understanding and modeling this axis with adequate models will be crucial for unraveling the complexity of pLGG pathogenesis and identifying novel therapeutic targets.

## MAPK pathway in pediatric low-grade gliomas

**Fig. 1:**
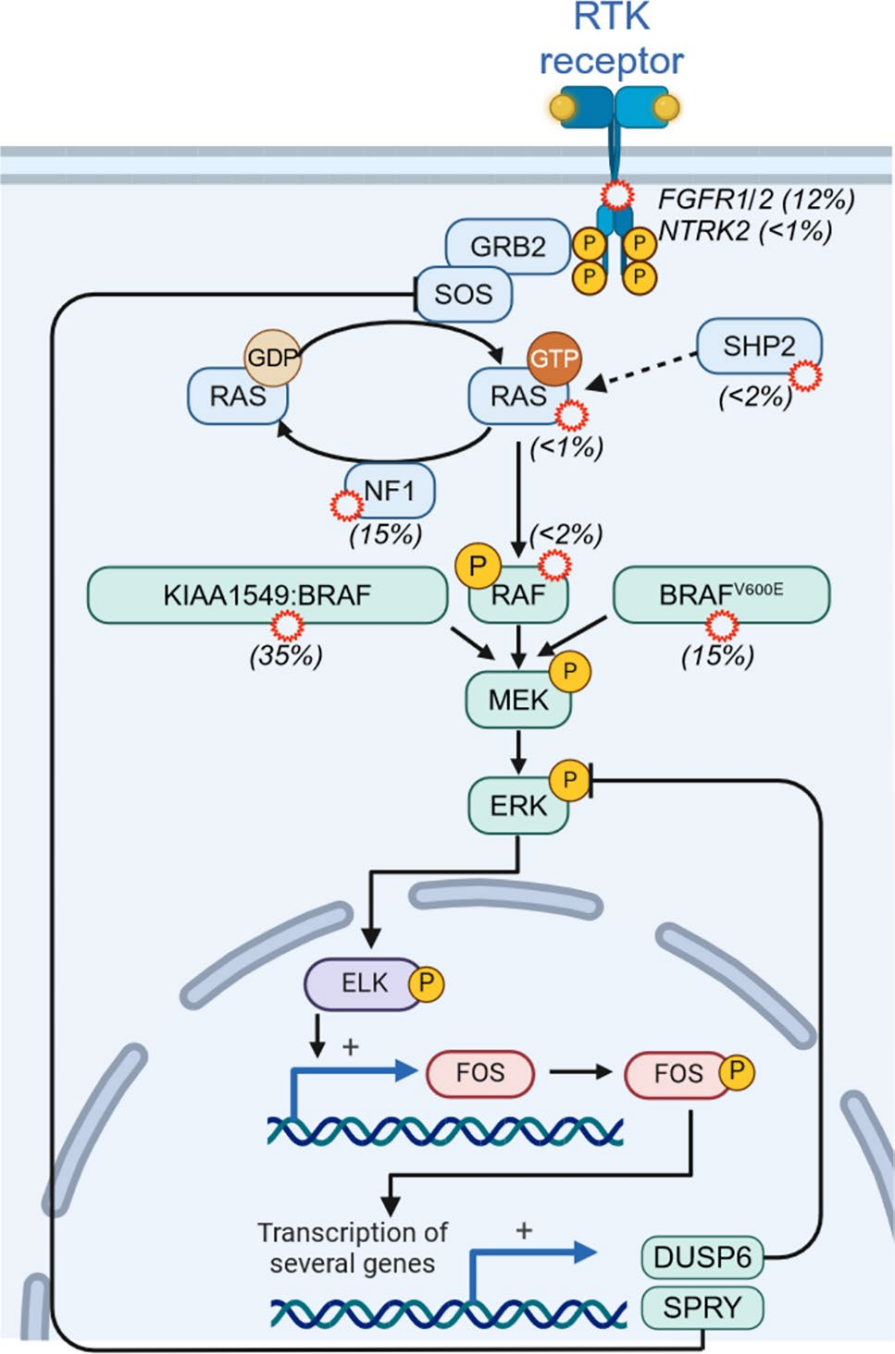
MAPK pathway in pediatric low-grade gliomas. RTK, receptor tyrosine kinase. Percentages from [7]

MAPK signaling pathways relay various extracellular signals, such as the presence of growth factors or stress, to cytoplasmic processes and transcriptional programs [9]. The RAS/RAF/MEK/ERK MAPK pathway plays not only a role in many physiological processes but is also dysregulated by somatic and germ-line mutations in tumor diseases and RASopathies, respectively [10]. pLGGs are a prime example for tumor diseases that are predominantly driven by aberrant ERK/MAPK activity due to mutations in core and accessory signaling elements of this pathway [6] (Fig. 1).

This signaling axis becomes activated by a plethora of cell surface receptors, with receptor tyrosine kinases (RTKs) being the most relevant in the context of this review. In pLGGs, alterations were identified in the RTKs NTRK2 and FGFR1/2 (< 1% and 12% of all pLGG, respectively) [7, 11]. RTKs are activated by growth factors inducing their dimerization, activation, and trans-phosphorylation resulting in the recruitment of effector protein complexes such as the Grb2/SOS complex promoting RAS-GTP loading [12].

The antagonistic reaction, the stimulation of GTP hydrolysis by RAS-GAPs such as neurofibromin 1 (NF1), is of particular interest from the pLGG perspective, as this tumor suppressor product is often lost or inactive in optic pathway glioma (OPG), which often occur in patients suffering from the RASopathy neurofibromatosis I (15% of all pLGG) [7, 13]. Albeit still mechanistically ill-defined, the proto-oncogene product SHP2/PTPN11 enhances RAS signaling, probably by multiple mechanisms [14]. Similar to NF1, gain-of-function SHP2 mutations are also found in LGG (< 2% of all pLGG) [7, 10]. Lastly, mutations affecting the intrinsic GTPase activity of RAS proteins are also found in pLGGs (< 1% of all pLGG) [7, 11].

Active RAS-GTP undergoes a conformational change resulting in the exposure of its effector loop and the recruitment of proteins with RAS binding domains such as the RAF proteins [15, 16].

pLGG is a tumor entity with a high frequency and diverse spectrum of RAF family kinase alterations [17]. Indeed, *KIAA1549::BRAF* fusion can be classified as the genetic signature alteration in pLGG (35% of all pLGG), and other BRAF fusions involving other genes have been reported [7, 18]. Almost all oncogenic BRAF fusion proteins reported so far share the inability to attain a closed inactive conformation as their pathomechanism [19]. As a result, these proteins bypass the need for the RAS-induced conformational change exposing the kinase domain and thus become strong MEK/ERK pathway activators due to their increased dimerization. Likewise, oncogenic RAF1 fusions have also been described in LGG, albeit at lower frequency (< 2% of all pLGG) [7, 18, 20–22]. However, RAF fusions are not completely uncoupled from regulatory layers [23, 24], unlike their BRAF^V600E^ counterpart, the second most common RAF alteration in pLGG (15% of all pLGG) [7]. Mechanistically, the glutamic acid residue from the BRAF^V600E^ mutation locks the BRAF kinase domain in an active conformation by mimicking the usual conformation change induced by its phosphorylation [25]. While most molecules form in fact stable homo-dimers as shown by complementary approaches [26–28], BRAF^V600E^ also exists in monomeric state.

In turns, the MAPK ERK1 and ERK2 phosphorylate hundreds of substrates [29]. ERK is well known to influence transcription by phosphorylating transcription factors such as ELK1, in turn inducing the transcription of so-called immediate early genes such as *FOS*, which encodes another transcription factor that initiates the second wave of transcription. Among these target genes, we find genes involved in negative feedback loops (such as DUSPs and SPRY proteins) [30], allowing for fine tuning of the MAPK pathway’s activity.

Despite the diverse spectrum of alterations affecting the MAPK pathway and its excessive activation as the pathomechanism driving pLGG, these mechanistically different mutations generate distinct signaling outputs that can be further modulated by the position of the oncoprotein in the signaling network topology and its independence form positive and regulatory principles. For example, the high ERK pathway activity observed in BRAF^V600E^-driven tumors is explained by its independence from RAS-regulated events and its insensitivity against negative feedback loops [26, 31, 32], while these regulatory requirements still apply to RAF proteins in tumors driven by RTK alterations and probably by other upstream elements such as RAS or SHP2 [33]. Thus, depending on the molecular alteration, MAPK signaling output might be different. This highlights the need to appreciate a spectrum of aberrant MAPK activity that in turn address specific and sometimes contrasting gene expression programs such as proliferation, differentiation, or senescence that are demarcated by distinct quantitative and/or temporal thresholds. This concept was already discussed almost 30 years ago [34] and has been corroborated by studies showing how spatio-temporal and quantitative differences in MAPK activity contribute to cellular decision making [35]. Even within pLGGs, there appears to be a preference for BRAF^V600E^ in supratentorial neoplasms, while cerebellar tumors are dominated by BRAF fusions [36]. This finding could also support the “goldilocks” hypothesis according to which certain pLGG precursor cells have different transformation thresholds, i.e. a critical amount of a “MAPK dose” that keeps the cells in cycle without causing either a resting state due to a too weak signal, or cell cycle arrest caused by a too high-dose triggering oncogene-induced senescence (OIS). Likewise, understanding the intrinsic factors, e.g. precise type of mutation or fusion partner, and extrinsic factors, e.g. the state of the intra- and intercellular signaling network, will be key to understanding the initiation of pLGG and their goldilocks principle driving tumor growth. Moreover, answering these questions will be key for a successful targeted therapy as an incomplete inhibition of a highly active BRAF oncoprotein triggering OIS instead of cell death in the majority of tumor cells might allow cell cycle re-entry and tumor expansion instead.

## Oncogene-induced senescence (OIS) and senescence-associated secretory phenotype (SASP) in pLGG

**Fig. 2:**
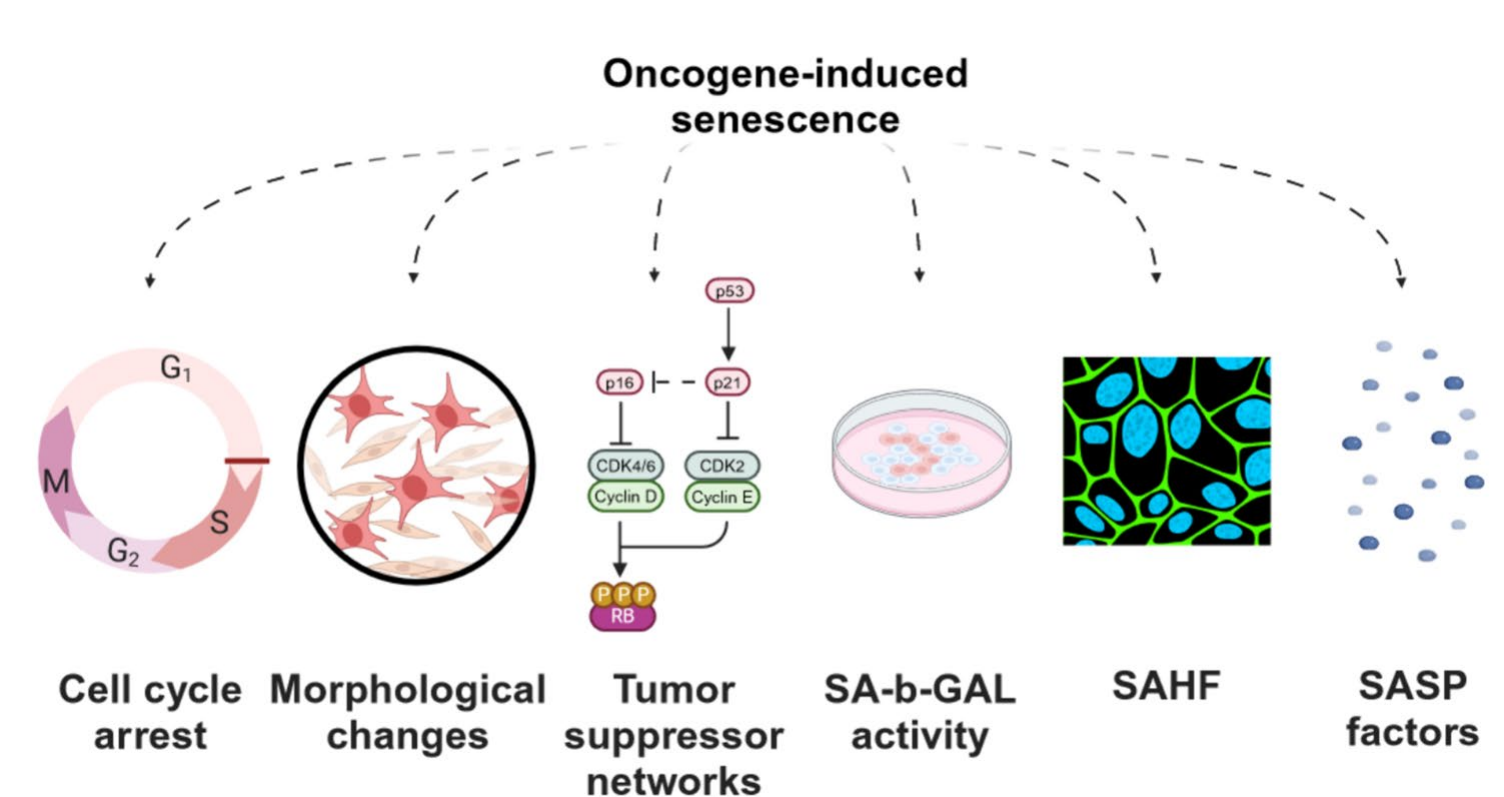
Molecular and cellular features of oncogene-induced senescence. SA-b-GAL, senescence-associated β-galactosidase; SAHF, senescence-associated heterochromatin foci; SASP, senescence-associated secretory phenotype

Among the many cellular processes regulated by the MAPK pathway, OIS represents one of the most characteristic of pLGG. Cellular senescence is characterized by loss of proliferative capacity with preservation of viability and metabolic activity [37]. Continuous attrition of telomers triggering DNA damage response pathways was identified as the causative molecular correlate of the observed phenotype, corresponding to replicative senescence [38]. In more general terms, however, senescence can also be triggered by various other stimuli including overactivation of oncogenes in cells with intact tumor suppressor pathways, leading to OIS [39]. Both types of senescence share a number of features including cell cycle arrest, morphological transformation (large and flat phenotype), activation of tumor suppressor networks (p53/p21 and p16/RB pathways), SA-b-GAL activity, and senescence-associated heterochromatin foci (SAHF) [40] (Fig. 2). However, a key difference between replicative senescence and OIS is that OIS is independent of telomere shortening and therefore cannot be bypassed by expression of telomerase [41].

Pilocytic astrocytoma (PA), the most common pLGG subtype [2], shows typical features of senescence [42], and patient-derived PA cell lines exhibit a typical OIS and not replicative [43] senescent phenotype with upregulation of senescence gene-sets [43, 44]. In line with the observation that OIS can be induced by overactivation of an oncogene in the context of an otherwise intact genome, oncogenic MAPK signaling driven by PA-typical MAPK alterations (e.g. BRAF V600E mutation; *KIAA1549::BRAF* fusion) is able to induce senescence in normal astrocytes and human neural stem cells in vitro [42, 45]. The prominent role of intact tumor suppressor pathways for the execution of OIS in pLGG is underlined by two observations: firstly, pleomorphic xanthoastrocytoma cells that harbor a deletion of the tumor suppressor gene *CDKN2A* encoding p16 do not go into OIS [46]; second, senescence can be bypassed by expression of SV40 large T antigen, which inhibits p53/p21 as well as p16/RB pathways in PA cells [43, 44].

OIS requires the senescence-associated secretory phenotype (SASP), a complex inflammatory network composed of several chemokines and cytokines secreted by senescent cells [47]. These factors maintain growth arrest (human fibroblast models of OIS) and attract immune cells leading to clearance of growth-arrested genetically altered cells (hepatocellular carcinoma) [47, 48] or conversely execute proangiogenic effects leading to formation and growth of tumors (e.g. epithelial cells and breast cancer cells) [49, 50]. While it is not established whether SASP factor expression is under the control of the MAPK pathway in pLGG, they are upregulated in primary human pLGG tumors as well as in senescent patient-derived PA cells, with IL1B and IL6 being the most important cytokines, inducing and maintaining OIS via NFkB activation in an autocrine manner [51].

OIS, representing a powerful cell-intrinsic fail-safe mechanism, may explain why patients with PA tumors experience slow tumor growth, no progression to higher-grade astrocytomas, and excellent overall survival rates [42, 45]. High expression of OIS [43] and SASP [51] gene-signatures in primary PA tumors correlate with excellent event free survival, suggesting a tumor controlling role of OIS. However, non-proliferating tumor cells in OIS are likely not responsive to anti-proliferative treatments and may constitute a source of tumor progression after discontinuation of anti-proliferative treatments [52]. OIS is therefore a novel as well as clinically yet unexploited molecular target for the improved treatment of pLGG.

## Tumor microenvironment (TME) in pLGG

While MAPK-driven pLGG cells characterized by OIS/SASP features represent an estimate of 50–60% of the bulk tumor, the remaining 40–50% are represented by microenvironmental cells [53]. Indeed, the TME is an important biological and clinical feature of brain tumors, plays critical roles in their development and progression, and has garnered clinical interest for therapeutic approaches [54]. Across brain tumors, the most common cell type found in the TME are myeloid cells (i.e. microglia and macrophages) [54–62]. In pediatric brain tumors, the amount and composition of the TME vary across tumor diagnoses. In particular, the proportions of immune infiltration tend to decrease with increasing WHO grade, making pLGG immune hot tumors compared to their cold and higher grade counterpart [59].

**Fig. 3:**
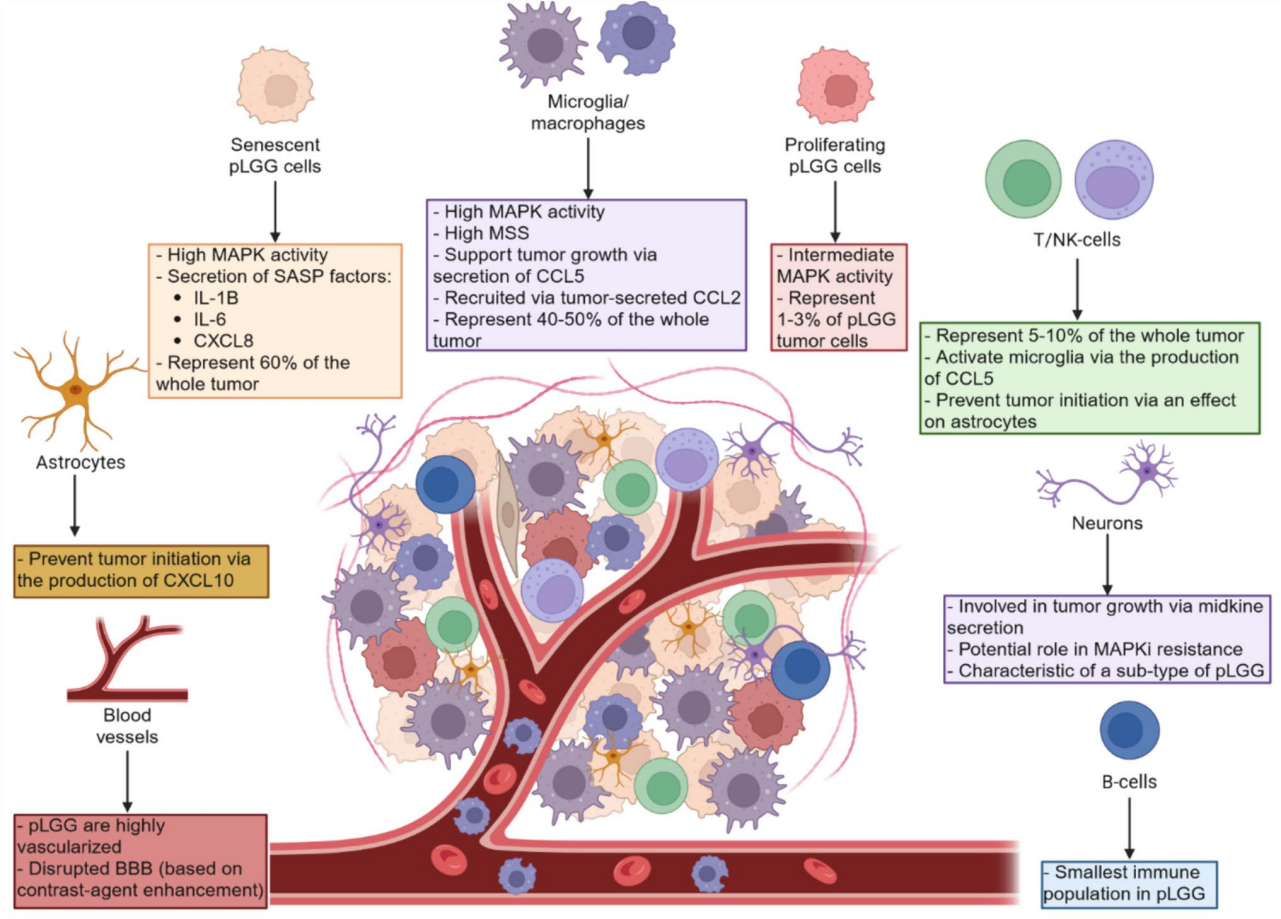
Tumor microenvironment composition and main features in pLGG. MSS, MAPKi sensitivity score; BBB, blood–brain barrier

The composition of the TME in pLGG is diverse and comprises many subpopulations (Fig. 3). The main non-malignant cell type found in pLGG tumors are microglia (30–50%) [53, 63, 64], followed by macrophages (~ 5–10%) [53]. While the debate how to clearly discriminate between macrophages and microglia in gliomas [65, 66] is still ongoing, these monocytic populations are characterized by a high expression of the surface markers CD68 and IBA1 [63, 64]. Of particular interest, MAPK activity was found to be high in pLGG-associated microglia [67], while proliferating microglia have been found to be enriched in pilocytic astrocytomas compared to astrocytomas of higher grade (III–IV), suggesting a major role of these cells in pLGG biology [68]. For instance, microglia cells are essential for the initiation of NF1-driven OPGs in a mouse model [69], while PFS is inversely correlated with proportions of CD68 + cells in PA [70].

Lymphocytes were also identified in primary pLGG samples, including CD3 + , CD4 + , CD8 + , and NK and Tregs cells (~ 5–10%) [53, 59, 71]. In particular, CD8 + T cells were found in higher fractions in PAs (3.28%) compared to other types of brain tumors (1.3–0.1%) [72], while the proportion of CD3 + T cells was shown to vary across pLGG subtypes, with PXAs (4.7%) and GGs (4.8%) showing higher content compared to PAs (0.9%) [71]. They are also characterized by a low expression of immune checkpoint protein PD-1 in PA (16.6% and 23.3% of all CD8 + and CD4 + T cells, respectively) [53, 72]. T cells also play an important role in tumor initiation and progression, as CD4 + T cells may inhibit tumor formation [73], while CD8 + T cells may promote tumor cell survival [74]. Of note, B cells were also identified in pLGG from deconvolution of methylation profiles and scRNAseq studies, however in small proportions (< 1%) [59, 67], making this population rather understudied in pLGG.

In addition to immune cells, some studies demonstrated a potential tumor growth-promoting role of neurons in pLGG. An integrated proteogenomics study showed two subgroups in pLGG, one characterized by gene signatures associated with immune and microglia activity, the second by signatures related to neuronal activity [75]. These data are in concordance with another pLGG multi-omics study showing an “immune hot” group, enriched for microglia and macrophages signatures, and a “neuronal” group, enriched for neuronal signatures [76]. A recent study showed that pLGG tumors with reduced MAPKi sensitivity scores (MSS) were of a neuronal type, possibly suggesting a role for neuronal features in MAPKi treatment resistance [67]. While glioneuronal synapses were observed in adult LGGs [77], a study of such structures in their pediatric counter-part remains to be pursued.

Finally, pLGGs are invaded by endothelial cells [59] and highly vascularized [78]. Vascularization plays an important role in tumor biology, and high microvessel density was associated with decreased PFS in optic pathway and hypothalamic gliomas [79]. In addition, PA were shown to have a rather disrupted blood–brain barrier [80], evident in the clinical observation of contrast agent enhancement. In line with increased VEGF expression in PA, similar to glioblastoma [78, 81], anti-VEGF therapy using bevacizumab has shown radiological and functional efficacy in pLGG patients during treatment [82–84]. Beyond this, molecular mechanisms underlying the interactions between tumor cells and the vasculature in pLGGs remain to be investigated.

## MAPK-OIS-SASP-TME axis: biology and potential clinical opportunities

**Fig. 4:**
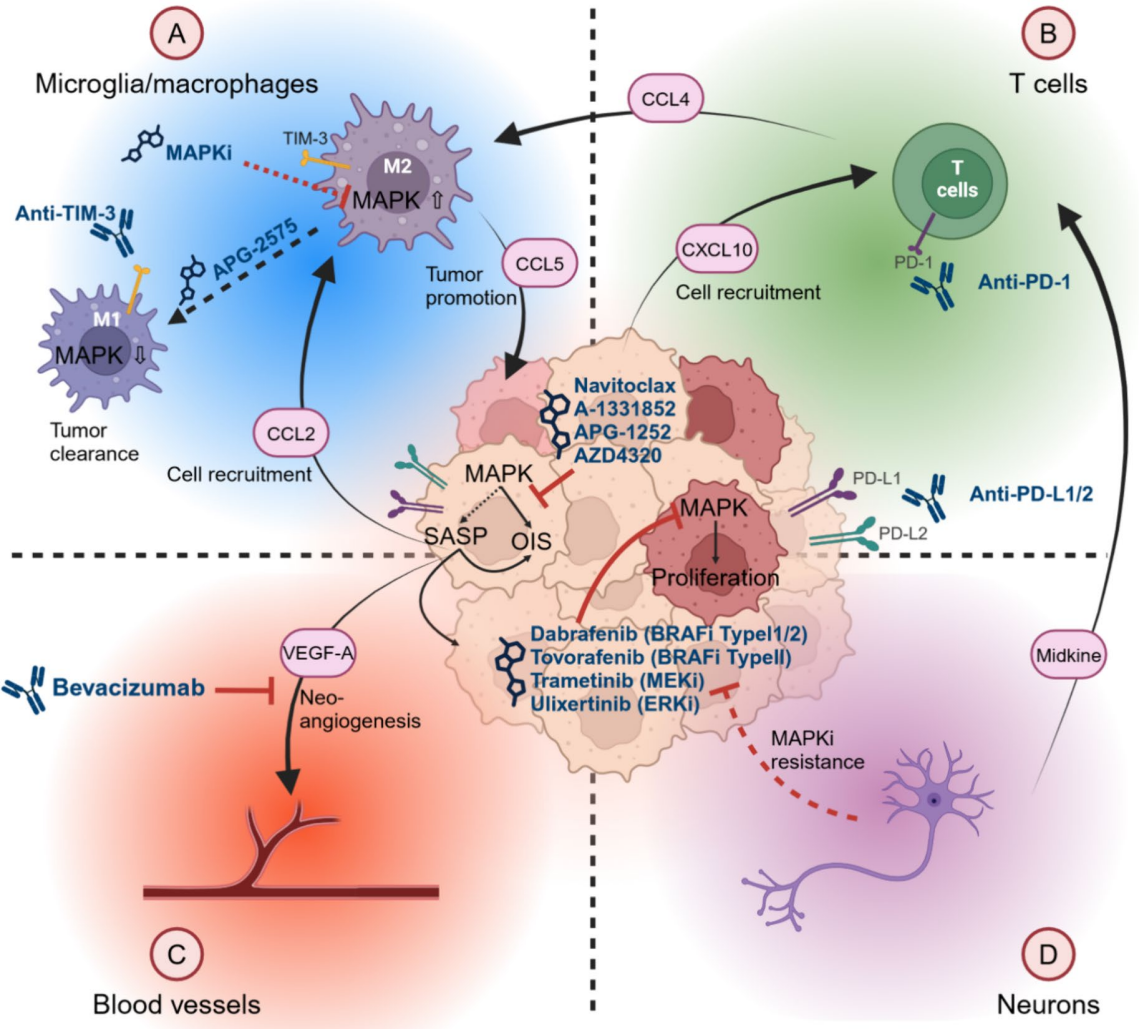
Integrated view of the MAPK/OIS/SASP/TME axis

While constitutive MAPK activation, induction of OIS/SASP and TME, represent more or less well-known key pLGG features on their own, their functional interconnection and cross-regulation has only recently become evident, and remains understudied. It has now become essential to envision these pLGG tumor components in an integrated manner, in what we propose to call the MAPK-OIS-SASP-TME (MOST) axis (Fig. 4).

The MAPK pathway plays in important intrinsic role in several brain TME cell populations. In microglia/macrophages, high MAPK activity was found to be associated with the polarization into a tumor promoting M2 phenotype, in adult glioblastoma (GBM) [85]. The MAPK pathway is also involved in the regulation of several processes in GBM-associated T cells, such as T cell proliferation, survival, activation, and aging [86–88], which can impair the anti-tumor immune response and favor tumor escape. The MAPK pathway is also highly involved in neo-vessel formation, as its effectors are found downstream of the main pro-angiogenic receptors VEGFR and FGFR [89], and can be activated during neo-angiogenesis in other tumor entities, such as renal and head and neck squamous cell carcinoma [90, 91]. Importantly, the MAPK pathway can promote an immunosuppressive environment in several preclinical models of colorectal cancer via the regulation of gene programs related to immune activity, such as interferon and inflammatory pathways, and reversible upon MAPK inhibition [87, 92, 93]. The MAPK pathway activity can also be dependent on the expression of the immune checkpoint protein TIM-3 in DIPGs, suggesting a role in the immuno-regulation of the TME [94]. While the MAPK pathway activity in the TME has been extensively studied in several entities, little is known about it in pLGG. Only recently a study found high MAPK pathway activity in microglia and macrophage populations in pLGG, associated with a high MSS [67]. Thus, these data indicate that the MAPK pathway plays a significant functional role in several populations of the TME of several tumor entities, including pLGG, possibly modulating the response to MAPKi treatment.

The OIS/SASP has a strong impact on the regulation of tumor development via its effect not only on the tumor cells, but also on the TME cells in pLGG. In a *KIAA1549::BRAF* fusion-driven neural stem cells (NSC) model, CCL2 secreted by the tumor cells, and under the control of the MAPK pathway, was necessary for the recruitment of microglia cells promoting tumor formation [95]. Increased secretion of cytokines by glioma cells upon MAPKi treatment and withdrawal was able to recruit microglia in an in vitro pLGG rebound model, highlighting the involvement of MAPKi signaling in the reshaping of pLGG TME via paracrine SASP factors (Kocher et al. in revision).

While SASP factors secreted by the tumor cells influence the pLGG TME, cytokines produced by TME cells can in turn influence the tumor cell compartment. For instance, CCL4 (secreted by CD8 + T cells) and CCL5 (secreted by microglia cells) were necessary to support OPG initiation via the induction of a neuronal-immune axis [74, 95]. Interestingly, CXCL10 produced by astrocytes was also important to prevent human-induced pluripotent stem cells (hiPSC)-LGG xenograft initiation via its activity on T cells [73].

SASP factors have been shown to be involved in the regulation of other TME populations in different tumor entities. VEGFA is generally involved in the induction of neo-angiogenesis [96]. The chemokine CXCL8 has been shown to be involved in the recruitment of neutrophils in colon carcinoma [97], while SASP gene expression signatures correlated with the expression of immune checkpoint proteins, such as PD-L1, in senescent fibroblast models [98]. It is important to note that senescent cells themselves can regulate their interaction with the immune system via reshaping their surface proteome and escape the clearance by the immune system, as has been shown in liver cancer [99]. Since these SASP factors are also produced by senescent pLGG cells, these mechanisms could be translated to pLGG.

All these findings, either demonstrated in pLGG or extrapolated from other entities and potentially transferable to pLGG biology, highlight the complexity of the MOST axis. All components influence each other forward and backward, in an auto- and paracrine manner, and in an extensive network of interactions and co-regulations. Therefore, an integrated analysis and understanding of pLGG and its TME can lead to new therapeutic strategies that target not only the tumor cells, but also the non-tumor cells and the interactions between these populations.

As an example, a combination of a MAPKi, potentially targeting the tumor cells and certain TME subpopulations, with an anti-angiogenic drug, such as bevacizumab, which on its own has already shown promising results in OPG in a retrospective study with regard to both tumor growth (at least stable disease was reached in 89% of patients) and visual function [82], could lead to a synergistic anti-tumor activity. Interestingly, similar progression patterns upon treatment withdrawal were observed in pLGG treated with either the VEGFRi bevacizumab [84] or MAPKi ([100], Kocher et al. in revision). Therefore, a sequential treatment regimen, with one treatment taking over when the other is withdrawn, could represent a way to prevent such progression upon treatment withdrawal.

As mentioned above, the MAPK pathway can promote an immunosuppressive microenvironment in other MAPK-driven entities [93]. Considering the importance of the MAPK pathway and immune cells in pLGG, a combination of MAPKi, inducing a more immunologically active TME, and immune checkpoint inhibitors, increasing immune cells’ activity, could potentiate the efficacy of the tumor cells’ clearance by the immune system. Such an approach, which already showed promising results in clinical trials in MAPK-driven CRC and melanoma [87, 93, 101], could be translated to pLGG.

Finally, senescent pLGG cells are dependent on pro-survival networks and can be targeted pharmacologically by senolytic drugs [44]. Senescent cells in pLGG, as well as other entities (melanoma, pancreatic carcinoma), can express PD-L1 [53, 98] or PD-L2 [53, 102]. These could be targeted by immune checkpoint inhibition facilitating the clearance of PD-L1/2 expressing senescent cells by the microenvironment, while inducing apoptosis in the remaining senescent cells with senolytic drugs. Preclinical data from lung adeno- and squamous cell carcinoma furthermore indicate that BCL-2 inhibitors such as APG-2575 were capable of reprogramming macrophages to a tumor suppressing M1 phenotype, thereby promoting the response to anti-PD-1 therapy [103]. This could represent a novel concept for pLGG treatment targeting the senescent compartment, with BCL-2 inhibitors both killing senescent tumor cells [44] as well as reprogramming the TME to promote PD-1 inhibitor response.

Taken together, the concept of the MOST axis illustrates the intricacies of pLGG tumors and opens the door to new therapeutic concepts to be investigated. While promising, a thorough pre-clinical evaluation of these treatment strategies will be necessary to support their future clinical investigations, highlighting the need for suitable preclinical models to model the MOST.

## Modeling of the MOST in preclinical models

Preclinical pLGG research relies heavily on the use of in vitro and in vivo models, limiting the scope of potential discoveries to the constraints of the respective models used. The general challenges and opportunities of available pLGG models are discussed elsewhere [104]. With respect to the MOST, many models are limited in the coverage of the complete MOST axis, and the selection of the appropriate model therefore needs be guided by the questions to be studied.

Thus, the ideal model retains the genetics, signaling pathway activations (MAPK and beyond), tumor and TME cell population proportions, and the communications between as well as the interdependencies of these populations, in order to study the full MOST axis.

**Fig. 5:**
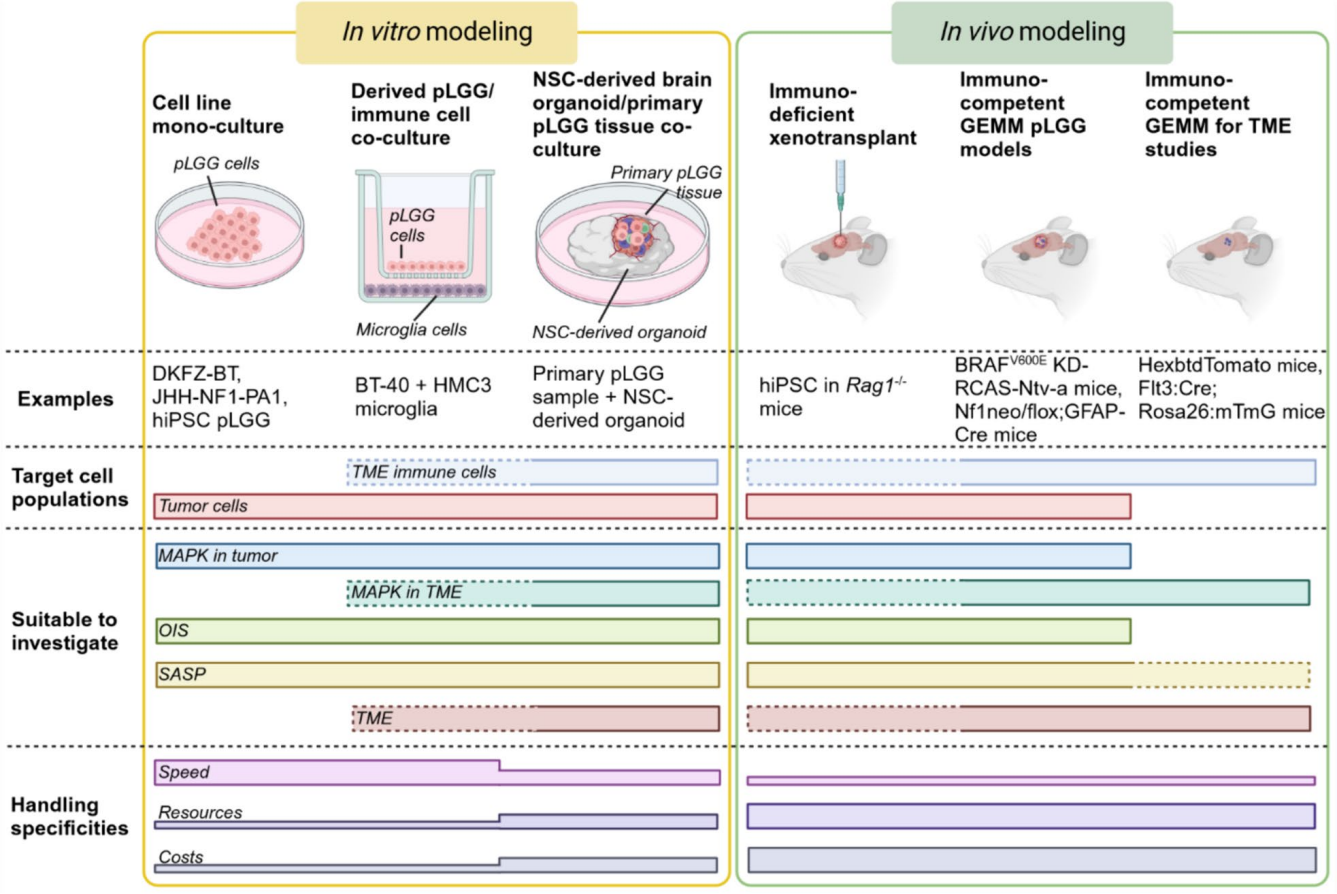
Overview of the MOST modeling strategies. NSC, neural stem cells; GEMM, genetically engineered mouse model; hiPSC, human-induced pluripotent stem cells

pLGG models currently available can be categorized in human primary tumor and hiPSC-derived in vitro cell lines and genetically engineered in vivo models [104]. pLGG in vitro cell line models derived from human primary tumors range from PA with a BRAF-fusion or -V600E mutation only (DKFZ-BT66, -BT308, -BT314, and -BT317) [43, 44] or *NF1* loss (JHH-NF1-PA1) [105], to pleomorphic xanthoastrocytoma (PXA) with BRAF^V600E^ mutation and *CDKN2A/B* deletion (BT40) [46]. hiPSC-derived pLGG cell lines harboring homozygous *NF1* loss or ectopic *KIAA1549::BRAF* expression have been developed for studies of progenitor cell biology in vitro and gliomagenesis as well as preclinical drug development [73]. While these cell line models offer pragmatic and substantial advantages in terms of scalability, long-term usability, prompt availability, and relatively modest expense, by themselves they completely lack cell population heterogeneity and TME interaction (Fig. 5). However, basic MOST aspects such as MAPK in tumor cells, OIS, and SASP can be easily studied in a high-throughput manner in these systems. To leverage the advantages of cell line cultures for the investigation of circumscribed MOST functions, defined co-cultures may be of advantage, such as pLGG cell line—HMC3 microglia co-cultures (Kocher et al. in revision). Neural stem cell (NSC)-derived organoid-primary tumor tissue co-cultures, as described for high-grade gliomas [106] and if transferable to pLGG, may be a future intermediate pLGG in vitro culture system, maintaining the primary tumor cells and the primary immune cell populations as well as possibly the tumor-intrinsic architecture within the TME.

Beyond in vitro cell culture, the hiPSC pLGG models are usable in vivo in immunodeficient *Rag1*^−/−^ mice [73]. While very well suited to study tumor cell-directed therapies in vivo, immunodeficient models lack intrinsic immune functions, limiting the scope of questions regarding the MOST axis. Immune-competent pLGG in vivo models comprise genetically engineered mouse models (GEMM) with *NfF1* loss [107–110] or ectopic BRAF^V600E^ kinase domain overexpression (BRAF^V600E^ KD-RCAS-Ntv-a) [111], as well as genetically engineered minipigs with *NFf1* loss [112]. Finally, GEMM to study specific TME populations exist, e.g. for microglia (i.e. tissue-resident macrophages) (HexbtdTomato mice) [113] and bone marrow-derived macrophages (BMDM) (Flt3:Cre; Rosa26:mTmG mice) [114, 115], opening the possibility to investigate consequences of MAPK inhibition on specific TME populations. In summary, immune competent pLGG GEMM, such as the BRAF^V600E^ KD-RCAS-Ntv-a, modeling BRAF^V600E^ mutated PA, or the Nf1neo/flox;GFAP-Cre, modeling NF1-deficient OPG, in particular represent an optimal overlap of prerequisites to study all aspects of the MOST axis, limited only by financial, infrastructural and expertise resources (Fig. 5).

It is thus dependent on the specific question to be studied, which model is optimally suited for MOST axis investigations. While genetically faithful models, true to pLGG biology, are becoming more widely available, the community is still very far away from full availability of models reflecting the wide range of genetically defined pLGG entities as recognized by the WHO [116], highlighting the need for further pLGG model development.

## Conclusion and translational roadmap

In this exploration of pediatric low-grade glioma (pLGG) through the lens of the MOST axis, we present a complex network of interactions that drives tumor behavior and response to therapy. This integrated approach highlights not only the intricacies of pLGG tumor and TME biology, but also potential avenues for innovative therapeutic strategies. The dysregulation of the MAPK pathway, a cornerstone in pLGG initiation and development, represents a prime target for intervention. However, as the MAPK pathway is closely intertwined with all aspects of pLGG biology, it cannot be viewed in an isolated manner, and as such requires further investigation to elucidate its role in pLGG’s TME populations. Moreover, the dual roles of OIS and SASP in tumor control and progression underscore the necessity for treatments that can navigate the fine line between inhibiting tumor growth and avoiding the promotion of a tumor-supporting microenvironment. Finally, the immunomodulation of the TME represent a new therapeutic opportunity which remains to be investigated in pLGG.

The translation of these insights into clinical practice demands a refined development of preclinical models that accurately recapitulate the disease. Such models are crucial for testing novel therapeutic agents and combinations. The impact of the MOST on the natural course of disease, as well as on (conventional, novel, and future) treatment response (i.e. treatment efficacy), as well as treatment effects on the MOST axis itself, need to be studied pre-clinically to provide a rationale for translation in clinical trials. Finally, clinical trials need to encompass reverse-translation, to include tumor material collection at appropriate timepoints (e.g. before, and if the need for an operation arises for clinical reasons, ideally also during or after treatment) in clinical trial protocols to study the MOST in primary patient tumor material. Therefore, concerted collaborative studies in the pre-clinical, translational, and clinical space will be necessary for the development of therapies targeting the MOST that are both effective and minimize long-term sequelae for pLGG patients.

By embracing the intricacies of all aspects of pLGG biology and leveraging advanced preclinical models, the path is set for groundbreaking advancements in therapy and patient care, marking a new era in pediatric neuro-oncology.

## Data Availability

No datasets were generated or analysed during the current study.
